# STOP: Study, Treat, Observe, and Prevent Neglected Diseases of Poverty Act

**DOI:** 10.1371/journal.pntd.0008064

**Published:** 2020-02-27

**Authors:** Peter J. Hotez, Cory Booker

**Affiliations:** 1 Texas Children’s Hospital Center for Vaccine Development, Department of Pediatrics and Molecular Virology & Microbiology, National School of Tropical Medicine, Baylor College of Medicine, Houston, Texas, United States of America; 2 Hagler Institute for Advanced Study at Texas A&M University, College Station, Texas, United States of America; 3 Department of Biology, Baylor University, Waco, Texas, United States of America; 4 James A Baker III Institute of Public Policy, Rice University, Houston, Texas, United States of America; 5 United States Senator for the State of New Jersey, Washington DC, United States of America; National Institutes of Health, UNITED STATES

In December of 2017, Philip Alston, the United Nations Special Rapporteur on extreme poverty and human rights, undertook a mission to the United States. He investigated the depth and breadth of poverty in the US, based on findings that more than 18 million Americans live in extreme poverty, including 5.3 million Americans who live in “Third World conditions of absolute poverty” [1] presumably referring to those living below the World Bank poverty level of $1.90 USD per day. His visit included predominantly African American areas in rural Alabama that currently and historically suffer from poverty, where evidence of endemic human hookworm infection was previously found [2], and he concluded that the extremely poor in several key areas of the US suffer an astonishing lack of health care access [1]. Professor Alston cited the state of healthcare for the poor in the United States as a “violation of human rights” and a “socially destructive policy aimed at the poor” [1].

As a group, the poverty related neglected diseases, including the neglected tropical diseases (NTDs), likely represent some of America’s most glaring health disparities. More than a decade ago, one of us (PJH) called attention to the widespread but often neglected infections of poverty in the United States [3]. They included chronic and debilitating parasitic infections, such as Chagas disease, cysticercosis, toxocariasis and other soil-transmitted helminth infections, trichomoniasis, and typhus (Box 1) [3, 4]. Although typically thought of as diseases of developing countries, our studies found that at least 12 million Americans live with at least one neglected parasitic infection [4,5], with many living in the five states of the US Gulf Coast, including Alabama, but also Texas, Louisiana, Mississippi, and Florida [6]. They are conditions that both arise in poverty and exacerbate poverty because of their long-term disabling effects. The United States is not alone in terms of wealthy nations harboring hidden burdens of poverty and NTDs. Indeed, within a health framework recently designated as “blue marble health” (to distinguish it from traditional norms of global health), today most of the world’s NTDs actually affect the poor living amid wealth in the group of 20 nations, including the US [5].

**Box 1. The major neglected infections of poverty in the US**

Parasitic Infections

- Chagas disease
- Cysticercosis
- Hookworm infection
- Toxocariasis
- Toxoplasmosis
- Trichomoniasis

Bacterial and Viral Infections

- Dengue and other arbovirus infections
- Typhus

The finding that the poor living in G20 nations now account for most of the world’s NTDs has important public policy implications. Specifically, if the leaders of the G20 economies would focus their energies and resources on their most vulnerable, we could see the prevalence or incidence of some of the most common NTDs reduced by one-half or even two-thirds [4]. So far, poverty-related neglected diseases or NTDs has not risen to the agenda of any G20 summit [7], but there are opportunities for individual governments to address the diseases of their most vulnerable.

Within the blue marble health framework, one of us (CB) has introduced legislation to launch a new fight for combating poverty-related neglected diseases in the United States [8, 9]. In October of 2019, S.2675 was introduced in the US Senate (116^th^ Congress) to authorize the Secretary of Health and Human Services “to carry out activities relating to the neglected diseases of poverty” [8]. Known as the *Study*, *Treat*, *Observe*, *and Prevent Neglected Diseases of Poverty Act* or the *STOP Neglected Diseases of Poverty Act*, this is the first legislation of its kind to focus on addressing poverty-related neglected diseases within the United States [8].

Among its significant features, the bill includes authorizing language to address the specific neglected disease policy concerns first raised in 2008 [3]. These include expanded and enhanced national surveillance efforts to reveal the true prevalence and distribution of these conditions, and to provide adequate diagnostic tools and clinical algorithms for federally qualified health centers [9]. It creates centers of excellence for research and development for new control tools–drugs, diagnostics, and vaccines–to combat the neglected diseases of poverty in the United States. It also calls on the Secretary of the Department of Health and Human Services (HHS) to develop educational programs for healthcare providers, since most US trained physicians, nurses and other healthcare professionals are not trained to recognize, diagnose, manage, and treat neglected diseases of poverty [9]. Finally, the legislation calls for the establishment of an interagency task force to provide recommendations to the HHS Secretary and the US Congress for diagnosing, treating and preventing neglected diseases of poverty in the United States. The task force would consult with leading experts on these neglected diseases of poverty [9].

So far, the bill has been endorsed by major policy organizations and academic societies committed to addressing NTDs and related neglected diseases of poverty, including: the American Society of Tropical Medicine and Hygiene, the Center of Excellence for Chagas Disease, the Center for Rural Enterprise and Environmental Justice, the Drugs for Neglected Diseases Initiative (DNDi), the Global Health Council, the Global Health Technology Coalition (GHTC), and PATH.

Many of the high prevalence NTDs in the Americas were first introduced into North America through the Middle Passage of the Atlantic slave trade, and therefore represent living legacies of slavery [10, 11]. For too long, the neglected diseases of poverty have remained ignored in the United States, despite the US government’s ability to address this serious issue that impacts millions of lives. This legislation represents an important first step towards addressing these vast and unfair health disparities at home.
